# Effects of cigarette smoke exposure on pulmonary physiology, muscle strength and exercise capacity in a retrospective cohort with 30,000 subjects

**DOI:** 10.1371/journal.pone.0250957

**Published:** 2021-06-24

**Authors:** Adil Adatia, Mustafaa Wahab, Izza Shahid, Ali Moinuddin, Kieran J. Killian, Imran Satia

**Affiliations:** 1 Department of Medicine, McMaster University, Hamilton, Canada; 2 Firestone Institute for Respiratory Health, St Joseph’s Healthcare, Hamilton, Canada; Ospedale S. Corona, ITALY

## Abstract

**Background:**

The effects of long-term cigarette smoke exposure on pulmonary physiology and how those effects lead to reduced exercise capacity are not well established.

**Methods:**

We retrospectively analyzed the spirometry, single-breath gas transfer (DLCO), peripheral muscle strength, and maximum exercise capacity data in patients referred to McMaster University Medical Centre for cardiopulmonary exercise testing between 2000 and 2012.

**Results:**

29,441 subjects underwent CPET and had a recorded smoking history [58% male, mean age 51.1 years (S.D.±19.6), BMI 27.4 kg/m^2^(±5.8)]. 7081 (24%) were current or former smokers and were divided into 4 categories by packs years (mean ±S.D.): <10 (5.8±3.3), 10–20 (17.1±2.9), 20–30 (27.1±2.8), 30–40 (37.3±2.8), and >40 (53.9±12.8). Patients with greater cigarette smoke exposure had lower expiratory flow rates (FEV1, FEF50, FEF75, PEFR), DLCO, and maximum power output (MPO) during exercise. There was no association between smoke exposure and muscle strength. Modeling MPO (kpm/min) output as a function of demographic and physiologic variables showed that the data are well explained by muscle strength (kg), FEV1 (L), and DLCO (mmHg/min/mL) in similar magnitude (MPO = 42.7*Quads^0.34^*FEV1^0.34^ * DLCO^0.43^; r = 0.84).

**Conclusions:**

Long-term cigarette smoke exposure is associated with small airway narrowing and impaired diffusion capacity but not with peripheral muscle weakness. The effects of smoking, age, and gender on maximum power output are mediated by reductions in FEV1, muscle strength and DLCO. Exercise capacity in smokers may benefit from therapies targeting all 3 variables.

## Introduction

Chronic obstructive pulmonary disease (COPD) is one of the commonest causes of hospital admissions [1, 2] and a leading cause of death worldwide [3–6]. There is no cure for COPD, and acute exacerbations lead to hospital admissions and are a major contributor to health care expenditures [7–9]. The frequency of exacerbations and symptoms of dyspnea increases as the forced expiratory volume in 1 second (FEV1) decreases [10–13]. Clinical efficacy and drug registration studies lasting 12 months often show modest improvements in exacerbations and decline in forced expiratory volume in 1 second (FEV1) [14–18]. Despite these improvement in FEV1 and exacerbations, improvements in breathlessness and exercise capacity are modest [17, 19–21]. Long term prospective studies suggest the natural history of the disease intractable to pharmacological intervention with inhaled corticosteroids and/or bronchodilators [22–26] and absolute risk reductions are small with methodological issues identified [27, 28]. Understanding the full extent of the effects of smoking on respiratory and muscle physiology may provide some important insights.

Cigarette smoke exposure causes an accelerated decline in FEV1, eventually leading to airflow obstruction (FEV1/FVC<0.7). The structural abnormality leading to airflow obstruction and reductions in flow rates include emphysema, alone and together with a variety of inflammatory cells and mucus causing small airway narrowing [29–32].The increased rate of decline is not fully reversed by smoking cessation [33], indicating that even patients with minimal smoking histories are at risk of at risk symptomatic respiratory disease in the long-term.

Impairment in pulmonary diffusion capacity may precede the reduction in FEV1 and is associated with worse symptoms and poorer exercise tolerance [34]. However, the FEV1 is the most frequently performed physiological measurement to diagnose and monitor the disease. Quantitative measurements of small airways disease and emphysema are seldom performed routinely. A comprehensive assessment of other physiological measurements apart from the FEV1 resulting from smoking exposure is under appreciated.

The objective of this study was to investigate the consequences of increasing cigarette smoke exposure in a real-world population on measurements of lung volumes and flow rates, gas transfer, muscle strength, symptoms of breathlessness/leg fatigue, and maximum exercise capacity on a cycle ergometer.

## Methods

### Study design

We conducted a retrospective study of consecutive subjects referred for cardiopulmonary exercise testing (CPET) to McMaster University Medical Center in Ontario, Canada between 2000–2012 with a recorded smoking history. The clinical indications for CPET included: (1) assessment of myocardial ischemia; (2) evaluation of exercise-related symptoms, particularly exertional fatigue, dyspnea, and chest pain; (3) screening prior to exercise training; and (4) monitoring progress in exercise performance following treatment and cardiorespiratory rehabilitation.

### Study procedures

Prior to CPET, the indication for exercise testing was reviewed, current drug therapy was recorded, any previous history of myocardial infarction was noted, and cigarette smoke exposure was quantified in pack years for all patients using a standardized questionnaire. Every patient provided written consent for performing the test and for the data generated to be used for clinical audit and research purposes. We did not seek additional ethics approval as all data were anonymized before we received it, the data are presented in aggregate form, and a large proportion of the 29,441 subjects have either died or left the area.

Prior to CPET, spirometry (FEV1, FVC, PEFR, FEF 50 FEF 75, and PIFR), single breath DLCO (DLCO, VA and KCO), arterialized capillary blood gases, haemoglobin (Hb) and carboxyhaemoglobin (HbCO) were measured in accordance with ATS standards [35, 36]. The strength of three peripheral muscle groups was assessed during seated row, seated press (bench press), and knee extension (quadriceps). Measurements were made following maximum voluntary contractions against hydraulic resistance (Hydrafitness Industries, Belton, TX). Maximal inspiratory and expiratory pressure (MIP, MEP) were measured during a maximal volitional effort against an occluded airway at residual lung volume and total lung capacity. The elastic recoil of the lungs and chest wall contributed to the pressures generated. Glottic closure was prevented by a small leak in the system.

CPET was performed under physician supervision using an electrically braked cycle ergometer (Siemens Elema 370; Siemens, Solna, Sweden) with electrocardiographic monitoring. Standard operating procedures were followed including the use of defined criteria for stopping such as serious cardiac arrhythmias, hypotension, and electrocardiographic changes, though termination of the test was rarely required. Before exercise, while seated comfortably on the cycle ergometer, subjects maintained tidal breathing for 1 minute and resting oxygen uptake was measured. Then subjects cycled at 60 rpm at an initial power output of 100 kpm/min, and the power output was incremented successively by 100 kpm/min every minute.

During exercise, oxygen uptake (VO2), carbon dioxide output (VCO2), respiratory quotient (RQ), heart rate (HR), blood pressure (BP), electrocardiogram (ECG), ventilation (VE), tidal volume (VT), respiratory rate (RR), end tidal and mixed expired carbon dioxide (PetCO2, PeCO2) and oxygen saturation (SaO2) were measured each minute following each increase in power output until symptom limited capacity.

Maximal power output was defined as the highest power output maintained for at least 30 seconds. During exercise, subjects were asked to estimate the intensity of leg effort, the intensity of the effort and discomfort required to breathe, and the intensity of chest pain every minute by matching their subjective estimate to simple descriptive phrases: just noticeable, very slight, slight, moderate, somewhat severe, severe, very severe, almost maximal, and maximal. The phrases were mapped to numbers from 0 to 10 to allow quantitative analysis (modified Borg scale) (13).

### Statistical analysis

The demographic data of the cohort were summarized using means and standard deviation (S.D.). Cigarette smoke exposure measured in pack years was binned using the categories: never smoker, <10, 10–20, 20–30, 30–40 and >40 pack years. The probability of self-reported myocardial infarction and measured airflow limitation was determined by dividing the number of patients with each of those factors within each cigarette smoke exposure category by the total number of subjects in that category. Lung volumes and flow rates, gas exchange at rest and during exercise, MPO, and VO2 max were analyzed using one-way ANOVA. Perceived intensity of leg and breathing effort (dyspnea) at rest and at each power output until MPO were assessed using MANOVA.

Physiological parameters that contribute to MPO were assessed by modeling MPO as a function of quadriceps strength, FEV1, DLCO, alveolar lung volume, KCO, age, height, weight, gender and cigarette smoke exposure. Multiple additive linear regression and non-linear interactive models using the same variables were explored. The large size of the dataset allowed age, height, weight and sex to be included in lieu of expressing measures such as FEV1 and alveolar lung volume as a percent of predicted normal values. For any given analysis, all subjects with measured values were used and those with missing data were excluded. All analyses were performed using TIBCO Statistica (Academic Package v13.2).

## Results

In total, 29,441 subjects underwent CPET from 2000–2012 and had a recorded smoking history. The demographics of this population are described in Table 1. Of the 29,441 subjects, 76% were never smokers reflecting the nature of referrals to our center, which routinely assesses patients with congenital heart disease, cystic fibrosis, and other non-smoking related conditions. An increase in the number of pack years smoked was associated with an increase in age, greater proportion of males, and a reduction in the maximum power output.

**Table 1:** Demographics of study population.

Smoke exposure Category (pack years)	Never smokers	<10	10–20	20–30	30–40	>40	All subjects
**n**	22360	2446	1785	1302	908	640	29441
**% Male**	55.3	59.3	65.3	69.7	73.0	76.6	57.9
**Age (years) ± SD**	49.1 ± 20.4	51.5 ± 17.8	57.6 ± 13.7	60.1 ± 12.0	62.1 ± 9.8	67.3 ± 9.6	51.1 ± 19.6
**BMI (m/kg^2^) ± SD**	27.0 ± 5.8	28.2 ± 5.4	29.1 ± 5.8	29.3 ± 5.4	29.5 ± 5.5	29.1 ± 5.6	27.4 ± 5.8
**Mean pack years ± SD**	0	5.8 ± 3.3	17.1 ± 2.9	27.1 ± 2.8	37.3 ± 2.8	53.9 ± 12.8	5.0 ± 11.8
**Maximum power output (kpm/min) ± SD**	825.9 ± 349.9	857.9 ± 334.2	802.9 ± 316.0	762.8 ± 297.6	678.0 ± 267.6	580.9 ± 268.0	814.5 ± 343.8

### Effect of smoking on probability of myocardial infarction and airflow limitation

**Fig 1:**
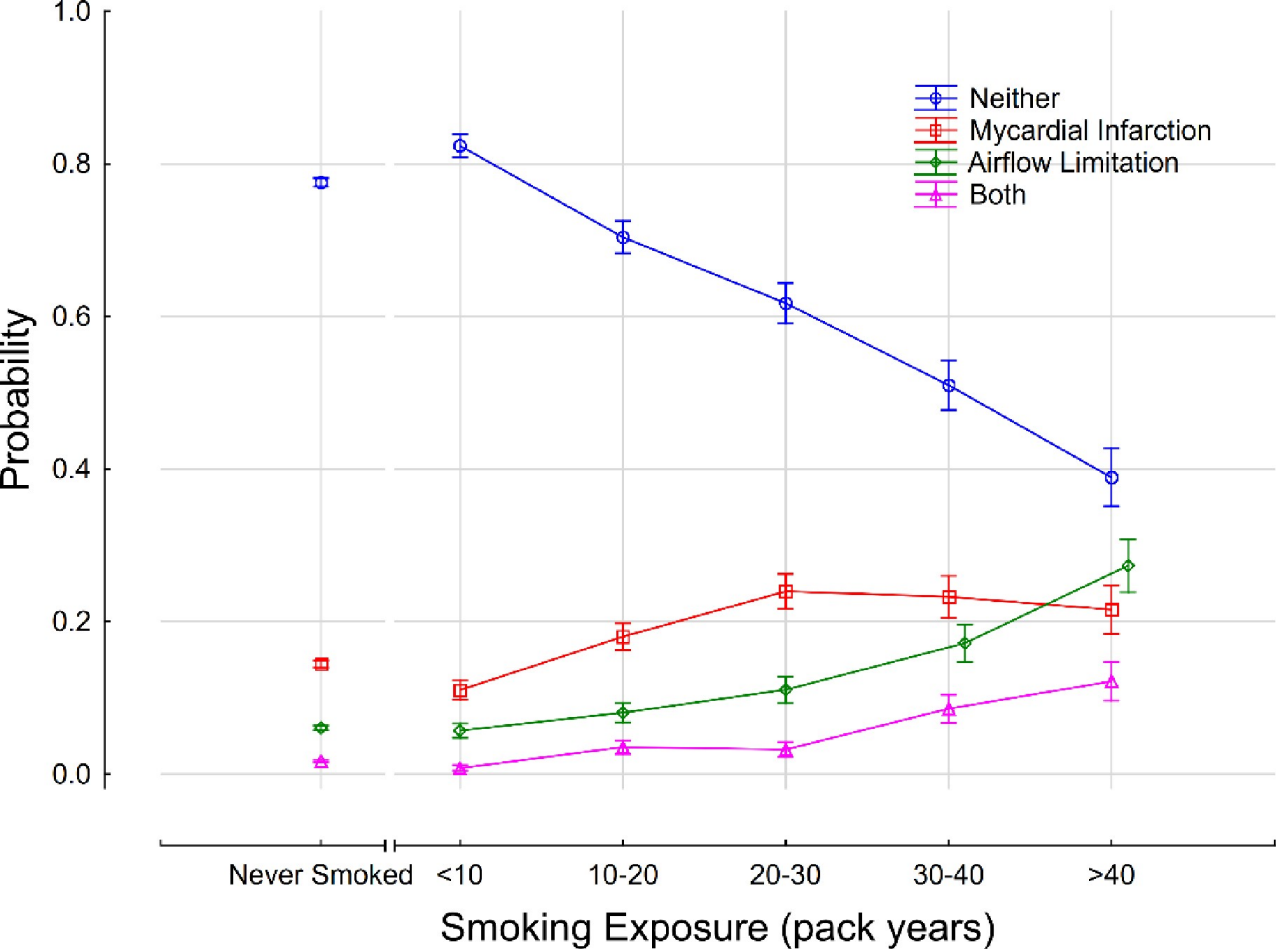
Effect of cigarette smoke exposure on the probability of self-reported myocardial infarction and measured airflow limitation. The probability of a subject reporting a history of a myocardial infarction and having measured airflow limitation is depicted by the purple line; the red line represents the probability of reporting a myocardial infarction with or without measured airflow limitation; and the green line represents the probability of measured airflow limitation with or without a history of myocardial infarction.

The probability of a self-reported history of myocardial infarction and measured airflow limitation is shown in Fig 1. The proportion of patients reporting a previous history of myocardial infarction increased with increasing cigarette smoke exposure from <10 to 20–30 pack years after which the proportion of patients remained similar. Similarly, there was an increase in the proportion of subjects with airflow limitation with increasing cigarette smoke exposure. In the >40 pack year category, only 38.9% (95% CI: 35.1, 42.7) of patients did not have a history of myocardial infarction or measured airflow limitation.

### Effect of smoking on lung volume and flow rates

**Fig 2:**
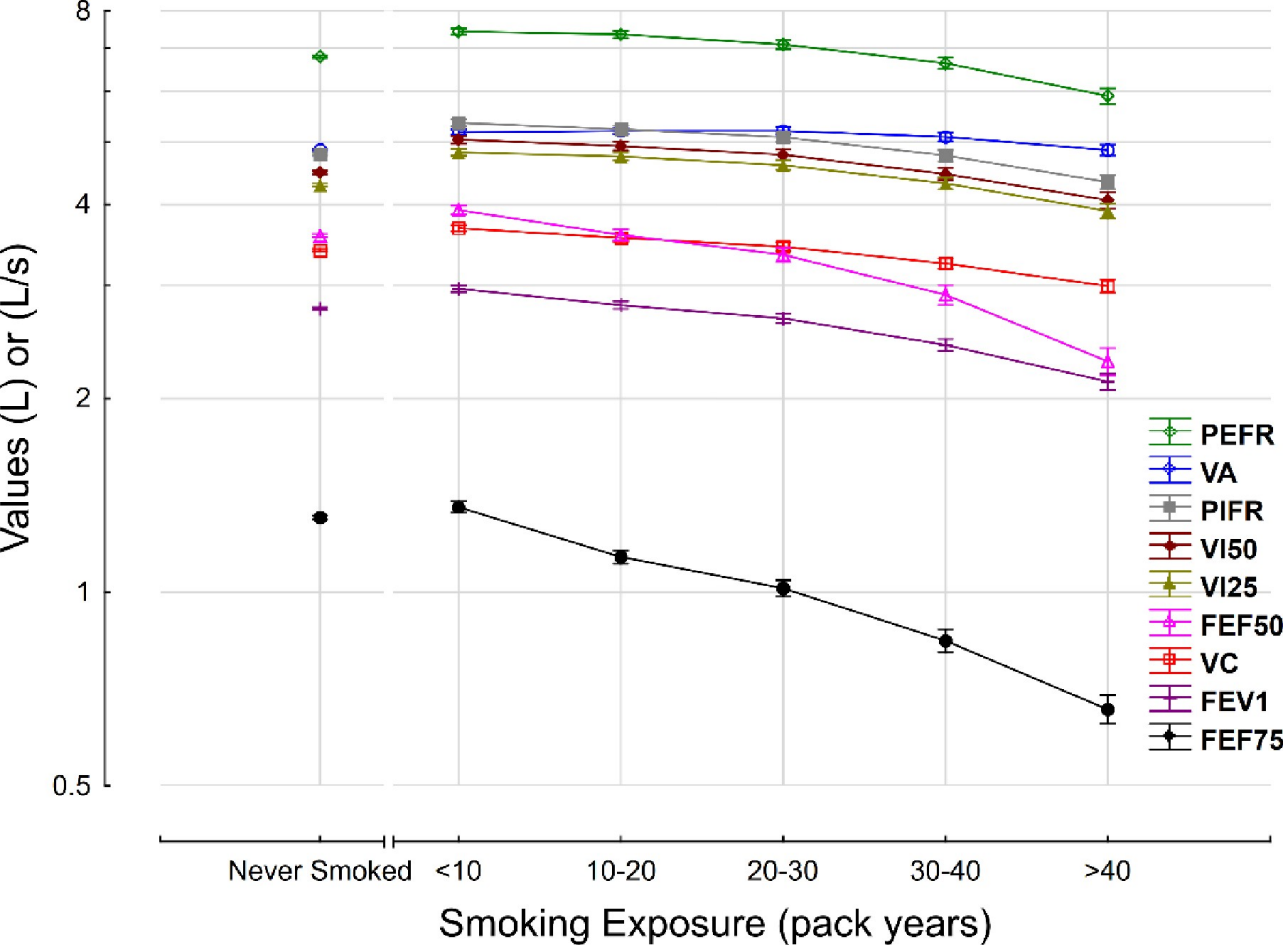
Effect of cigarette smoke exposure on lung volumes and flow rates. The y-axis is represented using logarithmic scale with base 2. FEF50; forced expiratory flow at 50%, FEF75; forced expiratory flow rate at 75%, FEV1; forced expiratory volume in 1 second, VA; alveolar volume, VC; vital capacity, VI25; inspiratory flow rate at 25%, VI50; inspiratory flow rate at 50%, PEFR; peak expiratory flow rate, PIFR; peak inspiratory flow rate.

Increasing cigarette smoke exposure was associated with a decrease in varying proportions of lung volumes and inspiratory and expiratory flow rates (Fig 2). Communicating alveolar volume (VA) remained relatively constant with greater smoke exposure, but vital capacity (VC) decreased significantly. The mean VA decreased by 6% from 5.2L (5.1, 5.2) to 4.9L (4.8, 4.9), whereas the mean VC decreased by 19% from 3.7L (3.6, 3.7) to 3.0L (2.9, 3.1). Expiratory flow rates declined disproportionately compared to the decrease in communicating alveolar volume. For example, the mean PEFR in the >40 pack year group was reduced by 21% compared to the <10 pack year group. The decline in flow rates with increased smoke exposure was most pronounced at low lung volumes; the FEF75 was 51% lower in the >40 pack year group compared to the <10 pack year group.

### Effects of smoking on pulmonary gas exchange

**Fig 3:**
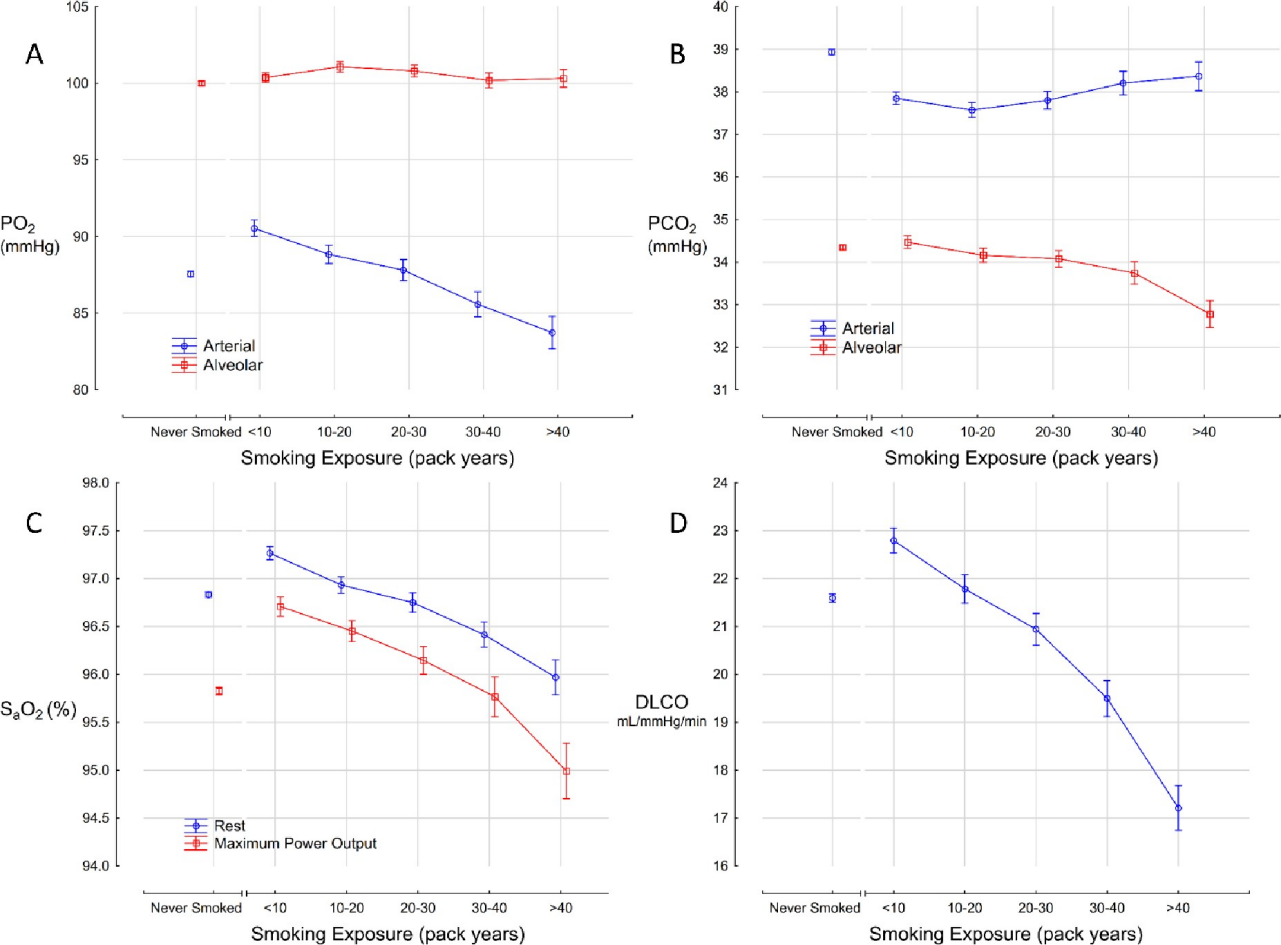
Effect of cigarette smoke exposure on the arterial-alveolar gradients for (A) oxygen and (B) carbon dioxide, (C) oxygen saturation, and (D) diffusion capacity.

**Fig 4:**
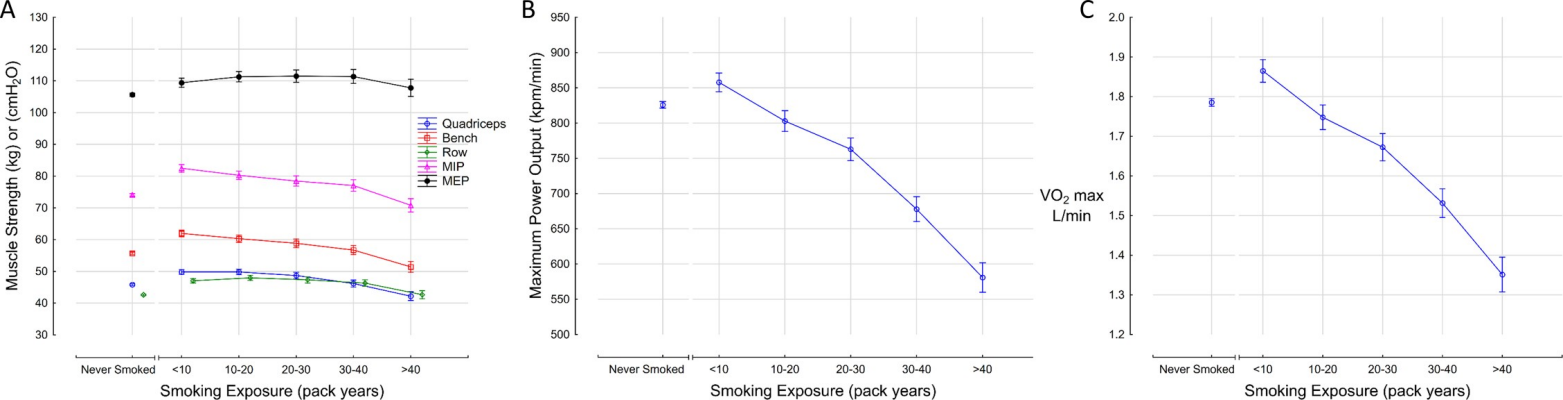
Effect of cigarette smoke exposure on muscle strength (A), maximum power output (B), and VO2 max (C). MEP; maximum expiratory pressure, MIP; maximum inspiratory pressure.

The arterial-alveolar gradient for oxygen and carbon dioxide are shown as a function of cigarette smoke exposure in Fig 3A and 3B. The partial pressure of alveolar oxygen (PAO2) remained similar across exposure groups, but the arterial oxygen pressure (PaO2) progressively decreased. In the <10 pack year group, the average alveolar oxygen tension was 90.5 mmHg (95% C.I. 90.0–91.1) and this decreased to 83.7 mmHg (82.7–84.8) in the >40 pack year group. Arterialized capillary blood sampling, direct measurement of end tidal samples, and conversion of SaO2 to PaO2 all yielded the same pattern (not shown). The alveolar-arterial carbon dioxide gradient increased with increasing smoke exposure, especially after smoke exposure was greater than 30 pack years (Fig 3B). Arterial oxygen saturation at rest and at maximum exercise declined as cigarette smoke exposure increased (Fig 3C). Finally, the proportionate decrease in DLCO declined the greatest as cigarette smoke exposure increases (Fig 4D). The mean DLCO in the >40 pack year group was 17.2 mL/mmHg/min (16.7, 17.7), which was 24% lower than the DLCO in the <10 pack year group, which was 22.8 mL/mmHg/min (22.5, 23.1).

### Effects of smoking on muscle strength, maximum power output, and VO2 max

The association of cigarette smoke exposure with muscle strength, maximum power output and VO2 max are shown in Fig 4A–4C, respectively. There was a modest decline in muscle strength in all muscle groups tested as smoke exposure increased. Mean quadriceps strength in the >40 pack year category, for example, was 42.2 kg (40.9, 43.6), compared to 49.8 (49.1, 50.6) in the <10 pack year group. There was a progressive decline in the capacity to generate power as cigarette smoke exposure increased and a parallel decline in VO2 max. The MPO and VO2 max were 29.7% and 24.3% lower, respectively, in the >40 pack year group compared to the <10 pack year group.

### Effects of smoking on perceived leg and breathing effort

**Fig 5:**
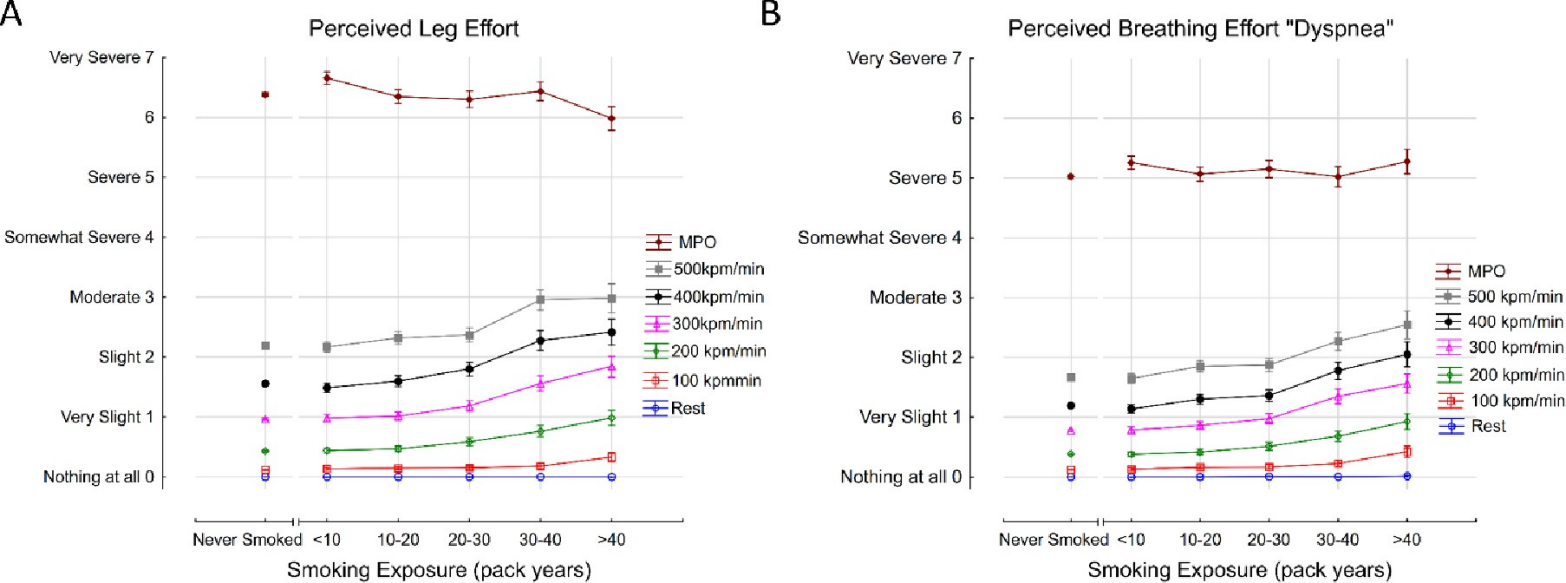
Association between cigarette smoke exposure and perceived leg (A) and breathing (B) effort during CPET. MPO; maximum power output.

The association between smoke exposure and the perceived leg and breathing effort is shown in Fig 5A and 5B, respectively. Subjects in each smoke exposure category terminated exercise after reaching similar symptom intensities for leg effort and dyspnea. Perceived symptom intensity increased with increasing power output and with increasing smoking exposure. At MPO, the intensity of leg effort was greater than the intensity of breathing effort in each smoke exposure category.

### Contributions to maximum power output

The power functions that best modelled MPO are shown in Eqs 1–5. The most important explanatory variable was quadriceps strength and using it as the only independent variable explained 50% of the variability in the data (Eq 1). Including FEV1 further improved the explanatory value of the model and including DLCO thereafter allowed for minimal improvement (Eqs 2 and 3, respectively). DLCO is predominantly determined by KCO and VA, and the substitution of DLCO for the measured KCO and VA did not improve the model (Eq 4). Finally, inclusion of cigarette smoke exposure expressed in pack years and age also did not further improve the model (Eq 5).


$$MPO\;kpm/min=54.4\;*\;Quads\;{\left(kg\right)}^{0.71}\;r=0.71$$ 1

$$MPO\;kpm/min=90.4\;*\;Quads\;{\left(kg\right)}^{0.42}\;*\;FEV1^{0.58}\;r=0.81$$ 2

$$MPO\;kpm/min=42.7\;*\;Quads\;{\left(kg\right)}^{0.34}\;*\;FEV1^{0.34\;*\;}DLCO^{0.43}\;r=0.84$$ 3

$$MPO\;kpm/min=42.7\;*\;Quads\;{\left(kg\right)}^{0.35}\;*\;FEV1^{0.36\;*\;}KCO^{0.44}\;*\;VA^{0.37}\;r=0.84$$ 4

$$MPO\;kpm/min=42.7\;*\;Quads\;{\left(kg\right)}^{0.35}\;*\;FEV1^{0.36\;*\;}KCO^{0.44}\;*\;VA^{0.37}\;*\;(1‐(0.025\;*\;pack\;years)(1‐(0.002\;*\;years>35)\;r=0.84$$ 5

## Discussion

This is the largest study to date investigating the association between long-term cigarette smoke exposure and exercise capacity, gas exchange, lung volumes and flow rates all in the context of one study. The primary novel findings of this study are 1) quadriceps strength, FEV1, KCO, and VA all contribute in similar proportions to maximum power output (MPO), 2) the reduction in MPO observed with increasing smoke exposure is mediated largely through impairment in KCO, VA, and FEV1, and 3) the effects of aging on MPO are also mediated through reductions in quadriceps strength, KCO, VA, and FEV1.

The adverse health effects of cigarette smoking have been thoroughly investigated since the 1950s, and the contribution of cigarette smoke exposure to coronary artery disease and airflow limitation is already firmly established [37]. Therefore, our finding that the probability of having airflow limitation and reporting a past myocardial infarction increased with increasing smoke exposure supports that our cohort is representative of the general population of patients undergoing cardiopulmonary assessment.

Increasing cigarette smoke exposure was associated with a decline in flow rates, with FEF75 more significantly affected compared to FEV1. Decreased FEF75 may indicate small airway disease, particularly in patients with a normal FEV1 [38], and thus this finding is consistent with existing data showing that early small airways disease may not be detected by FEV1 [39]. However, FEF75 is more variable than FEV1, dependent on the FVC, and has a large normal range, all of which preclude it from replacing FEV1 as the primary indicator of airflow limitation in smokers [38]. Other testing modalities, though, such as impulse oscillometry [40] and CT imaging in inspiration and expiration [41] may be useful for early identification of small airways disease in smokers.

The alveolar-arterial oxygen gradient increased with increased cigarette smoke exposure, despite preservation of the alveolar volume. Direct toxicity to the alveoli and disproportion of ventilation relative to perfusion across the alveoli undoubtedly contributed to this impairment and is reflected in the significant decline observed in DLCO. Oxygen uptake into the blood is both ventilation and perfusion limited in healthy individuals but becomes more diffusion limited when the surface area for gas exchange is significantly reduced, as seen in emphysema. This phenomenon becomes more pronounced during exercise due to the decreased transit time of blood through the pulmonary capillaries, which explains why the degree of oxygen desaturation at MPO increased with increasing smoke exposure.

There was a decrease in MPO and VO2 max observed in subjects with higher cigarette smoke exposure. Baseline quadriceps muscle strength, however, was relatively preserved. These findings indicate that the cardiopulmonary support of the peripheral muscles is impaired by smoke exposure rather than the strength of the muscles themselves, and this was associated with increased intensity of leg fatigue and dyspnea as smoke exposure increased. The intensity of effort at which exercise was terminated was similar on average between the smoke exposure categories, indicating that those with greater smoke exposure achieved lower MPO despite comparable effort. The association between smoking and muscle strength is unclear. Some studies have shown reduced muscle strength in smokers [42–48], but a meta-analysis that included 12 studies and data from more than 22,000 subjects found that the association between smoking and sarcopenia was inconclusive [49]. The importance of bone health is also often underappreciated as one study found lower levels of bone mineral content in smokers with obstructive lung disease, which negatively correlated with physical performance, particularly in women [50].

Modeling maximum power output as a function of muscle strength, ventilatory capacity (FEV1), communicating lung volume (VA) and gas transfer capacity (KCO) accounted for the majority of the variability in maximum power achieved. Muscle strength was the strongest predictor of maximum power followed by FEV1, VA, and KCO. The effects of height, weight, age, gender and cigarette smoke exposure are expressed through these factors given that their inclusion, in addition to the physiologic parameters above, failed to improve the model. Inclusion of cardiac function in the model may further improve its predictive capacity, but the ability to increase cardiac output cannot be independently assessed as the heart is dependent on the venous return (preload), which is a function of the power generated by the peripheral muscles.

These results have a number of important clinical implications. Reduced exercise capacity in those with significant smoke exposure is an important source of morbidity [51]. In addition to routine cardiopulmonary investigations, perhaps some patients whose degree of impairment is not adequately explained by those tests should have muscle strength quantified since it is an important contributor to power generation and a potential treatable trait. Bronchodilator therapy in COPD patients modestly improves breathlessness scores and exercise capacity [18–21], which is also consistent with our findings. For example, in an average individual smoker who has quadriceps strength of 35 kg, FEV1 of 2.7L, and a DLCO of 19.5 mL/mmHg/min, improvement in the FEV1 by 300 mL using bronchodilators would be expected to increase the MPO by only 3.9% using our model. Therefore, significant improvement in the exercise capacity of such patients may require a comprehensive treatment approach in addition to smoking cessation including bronchodilator therapy, muscle strengthening exercises, and perhaps future treatments aimed at improving gas transfer capacity. There is no medical intervention to improve gas transfer capacity apart from smoking cessation, which only partially reverses the impairment in DLCO [52]. Pulmonary rehabilitation has shown some benefits in exercise endurance [53–55], and one of the underlying physiologic mechanisms may be due to improved muscle strength.

This study has a number of important limitations. First, patients able to exercise are inherently healthier than those who cannot, so there is unavoidable selection bias present when studying the physiology of those referred for CPET. The results are thus not generalizable to the most severely affected patients with COPD or those with other comorbidities such as cardiac diseases that have contraindications to CPET. Second, the study was retrospective and patients were not followed longitudinally to assess the impact of smoke exposure as their number of pack years accumulated over time, both of which limit the strength of the conclusions. Third, the results were not separated by current smoking status. The decrease in DLCO partially resolves after smoking cessation owing to decreased carboxyhaemoglobin concentrations, and this could have influenced our DLCO data [52]. Fourth, we do not have data on the medication subjects were taking so were unable to investigate the effects of medical treatment.

## Conclusion

The primary adverse effects of long-term cigarette exposure on pulmonary physiology are small airway narrowing and impairment in alveolar diffusion capacity. FEV1 and DLCO contribute in equal proportion with peripheral muscle strength to determine MPO in long-term smokers. The effects of age, gender, and smoke exposure on MPO are mediated by deleterious effects on these three physiologic parameters.

## Abbreviations

CPET: cardiopulmonary exercise test
DLCO: diffusion capacity for carbon monoxide
FEF50: forced expiratory flow at 50%
FEF75: forced expiratory flow rate at 75%
FEV1: forced expiratory volume in 1 second
FVC: forced vital capacity
KCO: carbon monoxide transfer coefficient
MPO: maximum power output
VA: alveolar volume
VC: vital capacity
VI25: inspiratory flow rate at 25%
VI50: inspiratory flow rate at 50%
PEFR: peak expiratory flow rate
PIFR: peak inspiratory flow rate
